# Toxicity of Bisphenol in Pregnant Females: First Review of Literature in Humans

**DOI:** 10.7759/cureus.39168

**Published:** 2023-05-18

**Authors:** Radhika Agarwal, Shrirang S Joshi

**Affiliations:** 1 Physiology, All India Institute of Medical Sciences Rishikesh, Rishikesh, IND; 2 Emergency Medicine, All India Institute of Medical Sciences, New Delhi, IND

**Keywords:** unexplained recurrent pregnancy loss, plastics, environmental toxicology, systemic toxicity. allergy immunology. genotoxicity. reproductive toxicity. hazardous substances phthalate, bisphenol a

## Abstract

Bisphenol analogues are widely used in consumer products such as disposable dinnerware, canned food, personal care products, bottled beverages, and more, and dietary exposure is the main pathway. Bisphenol A is used to manufacture synthetic resins and commercial plastics in large quantities. According to epidemiological and animal studies, bisphenols disrupt the reproductive, immunological, and metabolic systems. These analogues are estrogenic like Bisphenol A, although human studies are limited.

We did a thorough search of the literature on the toxicity of bisphenol on reproductive and endocrine systems in pregnancy, focusing particularly on human studies. Hence, we present a comprehensive literature review on this topic.​​​​​​​ During our literature search, three epidemiological studies and one human observational study demonstrated a substantial link between bisphenol toxicity and recurrent miscarriages.​​​​​​​ The aforementioned research shows that bisphenol may harm pregnancy and cause miscarriages. We believe this is the first literature review on the topic.

## Introduction and background

Why worry about bisphenols?

Bisphenol analogues belong to the group of chemicals called diphenylmethanes, which have two phenol rings connected by one carbon atom with varying substituents [1]. Analogues of bisphenol are extensively used in the manufacturing of consumer products (e.g., disposable tableware, canned food, personal care products, bottled beverages, etc.), and dietary intake is regarded to be the primary route of exposure [2,3]. Concentrations range from nanograms to microliters per litre in ambient matrices, human fluids, and tissues [2,4,5]. Bisphenol AF (BPAF), bisphenol AP (BPAP), bisphenol S (BPS), bisphenol B (BPB), and bisphenol P (BPP) are the most utilized BPA-free substitutes. These counterparts possess the same chemical structure and physical and chemical properties as BPA. There have been questions raised about whether these chemicals could also disturb the endocrine system and have negative effects on reproductive health [6].

Bisphenol A (BPA) is a compound that is produced in massive quantities and is commonly utilised in the production of synthetic resins and commercial plastics. It is also present in a wide range of consumer goods [7,8]. Many epidemiological research and trials on animals have found BPA to be an endocrine disruptor that has negative effects on reproductive, immunological, and metabolic systems [9,10]. Since 2010, the European Union, Canada, France, the United States of America, and China have all enacted strict regulations that ban the incorporation of BPA in the manufacture of baby bottles [11]. Additionally, the usage of BPA in the production of thermal paper has been restricted since 2016 in the European Union; however, the complete ban did not go into effect until 2020 [12]. Government regulations and public concerns about BPA in recent years have encouraged the production and use of bisphenol analogues as substitutes. Due to the absence of regulations on these BPA-free analogues, these bisphenol equivalents are currently permissible to use without limitation [13,14]. Laboratory and animal experiments have proven these analogues to possess estrogenic activity comparable to or even stronger than BPA; however, human evidence is lacking [15-18].

## Review

Methods

We did a thorough online search of the literature on the harmful effects of bisphenol compounds on the reproductive and endocrine systems, focussing particularly on its toxic effects in pregnancy. Till March 2023, a number of studies showing the harmful effects of bisphenol in lower animals were conducted. Only a handful of research was found on the toxic effects of bisphenol on pregnancy, conducted in humans. Hence, we present a comprehensive literature review on the toxic effect of bisphenol on pregnancy in humans.

Results

Toxicity in Pregnancy

A tragic pregnancy outcome and a growing global issue, recurrent miscarriage (RM) is defined as two or more consecutive pregnancy losses before 20 weeks of gestation [19-21]. It can have significant consequences for females and their families. Approximately 2% to 5% of couples trying to get pregnant are affected by RM [22]. The cause of RM is intricate. At least 50% of women experience RM without a clear cause, often called unexplained RM (URM). No effective prevention is currently available for URM [23,24].

New research raises the possibility that pollution contributed to the increase in miscarriage rates [25-28]. Only a few research with relatively small sample sizes have previously examined this relationship, and the results too were conflicting. A small case-control study did not support the strong link between BPA exposure and an increased risk of URM that was suggested by two other case-control studies (odds ratio (OR): 3.91-9.34) [29-31]. Much less research has been conducted on the connection between BPA equivalents and URM. Only one cross-sectional study revealed a possible association between BPS, BPA, and URM [32].

Vulnerability of the Reproductive System

The reproductive system was shown to be vulnerable to endocrine-disrupting chemicals (EDCs), according to Uzumcu and Zachow, and Meeker [33,34]. Numerous epidemiological studies have linked the risk of URM to exposure to EDCs like phthalates, polychlorinated biphenyls, and dioxins in recent years [31,35,36]. These studies were conducted in countries all over the world. Being a typical EDC, BPA has been the source of grave worries regarding the potential effects it may have on URM.

Link Between RMs and Bisphenols

Three epidemiological studies in total found associations between BPA and URM. In a study conducted in Japan by Sugiura-Ogasawara et al., serum BPA levels were assessed in patients (n = 45) with a history of three or more recurrent first-trimester abortions and in healthy women (n = 32) with no history of live birth or infertility [29]. Since the blood BPA level in the cases was significantly higher than in the controls (P = 0.024), it suggests that exposure to BPA is related to URM. Similar findings were reached in large case-control research by Ao et al. [37]. According to a hospital-based case-control research in China, women with urine BPA levels between 0.40 and 0.93 g/g Cr and above 0.93 g/g Cr had a significantly increased chance of developing RM (odds ratio = 3.91; 95% confidence interval (CI): 1.23, 12.45 and OR = 9.34; 95% CI: 3.06 to 28.44). One hundred two patients who self-reported having a history of several, unexplained miscarriages were enrolled in the study [30]. Another case-control study conducted in China with a smaller sample size of only 60 people found no statistically significant changes in the levels of BPA found in the two groups (P > 0.05) [31]. The potential toxicity of BPA analogues on the foetus has recently attracted increasing attention, yet there is very little data to back this up. Only one case study, which included 111 URM patients, revealed any relationships between BPA and BPS levels, oxidative stress, and immunological homeostasis. The study’s authors postulated that there might be etiological and pathogenic connections between the biomarkers, bisphenol analogues, and URM [32]. According to current knowledge, there was only one study that focused on BPA mimics and URM at a wide scale (1,180 URM cases and 571 Controls) [37].

Effect of Bisphenols on the Hormonal System and Embryogenesis

Bisphenol analogues have been shown by Pollack et al. to mimic oestrogen or progesterone and interact with the oestrogen or progesterone receptor (ER or PR) to form complexes [38]. These analogues also form complexes with ERs and PRs. These latter molecules can, in turn, then adhere to the nuclear DNA response element, activating the downstream transcription factors, which subsequently initiate estrogenic effects. By acting on estrogen-related receptor 1 (ERR1), BPA was shown by Morice et al. to change the process of cell proliferation in human trophoblastic cells [39]. ER/PR-dependent signalling was found to be disturbed by BPA exposure in an in vivo study by Li et al., which in turn lowered epithelial receptivity and stromal cell decidualization in the early stages of pregnancy [40]. Researchers led by Tran et al. found that BPA has a detrimental impact on the implantation of embryos in mice due to its interference with growth and development factors mediated by PR [41]. Interfering with the hypothalamic-pituitary-gonadal (HPG) axis is one way that bisphenol analogues can affect the endocrine functions of the body. This can influence the synthesis, secretion, and release of hormones. The levels of plasma estradiol, luteinizing hormone, and follicle-stimulating hormone significantly increased after receiving BPA orally for six hours in adult female mice during proestrus, according to Wang et al.’s findings [42]. These results provide in vivo evidence that BPA exposure, even at low concentrations, can impair the HPG reproductive endocrine system.

Bisphenols Affect the Genetic Programming

URM is most frequently caused by an abnormal embryonic karyotype, which accounts for 50.3% of all cases [43]. Particularly prevalent is aneuploidy, which is detected in 29.9% of the cases [44]. There is evidence from various research suggesting that bisphenol analogues may interfere with the course of meiosis in oocytes. Human embryonic oocytes from cultured ovaries exposed to BPA displayed delayed pairing-synapsis and altered recombination processes during the meiotic prophase of the cell cycle, according to research by Brieno-Enrquez et al. [45]. Additionally, they discovered that BPA led to gene overexpression in cultures of human foetal ovarian tissue. According to Brieno-Enrquez et al., each of these genes had a role in the creation of double-strand breaks, the subsequent signalling, and the subsequent repair of those breaks during the meiotic development of female germ cells [46]. In spite of this, the effects of bisphenol analogues on URM as well as the underlying mechanisms are both in need of further investigation. According to research by Ao et al., women over the age of 30 were more likely than younger women to experience an association between bisphenol analogues and URM risks. It has been demonstrated beyond a reasonable doubt that the primary contributor to URM is embryonic aneuploidy. And the only etiological component that is thought to have an unquestionable connection to aneuploidy is a woman's age [47,48]. It is believed that the primary mechanism involved is an age-related change in the cohesiveness of the chromatin [49]. During meiosis, the cohesion of sister chromatids is preserved by a specific group of proteins that are referred to as cohesins. Recent studies have shown that in mouse oocytes, the amount of cohesin bound to chromosomes decreases with age. This finding revealed that the cohesin protein's deterioration is what causes the age-related increase in chromosomal segregation errors [50]. Brieno-Enrquez et al. found evidence that BPA may also interfere with the function of chromosomal cohesion (REC8) thus causing meiotic dysfunction [45]. Ao et al. hypothesised that older women might be more vulnerable to the harmful effects of the environment because of the heightened effect of bisphenol analogues on meiosis [37].

## Conclusions

Bottom line

Bisphenols are commonly used compounds in plastic manufacturing industries. Many bisphenol analogues have been found to have toxic effects on the reproductive and endocrine systems. Lately, there have been a number of studies to prove these toxic effects. It is evident from the above studies that bisphenol can indeed have a significantly detrimental effect on pregnancy and can lead to miscarriages. As per our recent knowledge, this is the first review of the literature regarding the topic.
